# NLRP3 Inflammasome in Vascular Disease: A Recurrent Villain to Combat Pharmacologically

**DOI:** 10.3390/antiox11020269

**Published:** 2022-01-29

**Authors:** Ainara González-Moro, Inés Valencia, Licia Shamoon, Carlos Félix Sánchez-Ferrer, Concepción Peiró, Fernando de la Cuesta

**Affiliations:** 1 Department of Pharmacology and Therapeutics, School of Medicine, Universidad Autónoma de Madrid, 28029 Madrid, Spain; fatima.gonzalez@uam.es (A.G.-M.); ines.valencia@uam.es (I.V.); licia.shamoon@uam.es (L.S.); carlosf.sanchezferrer@uam.es (C.F.S.-F.); concha.peiro@uam.es (C.P.); 2 PhD Programme in Pharmacology and Physiology, Doctoral School, Universidad Autónoma de Madrid, 28029 Madrid, Spain; 3 Instituto de Investigación Sanitaria del Hospital Universitario La Paz (IdiPAZ), 28029 Madrid, Spain

**Keywords:** NLRP3 inflammasome, IL-1β, IL-18, inflammaging, pharmacology, vascular, atherosclerosis, oxidative stress

## Abstract

Despite the great advances in medicine, mortality from cardiovascular diseases keeps on growing. This tendency is not likely to change considering the pandemic proportions of obesity and diabetes. Besides, the global population is more aged as life expectancy increases, and vascular aging plays a key role in the increased risk of vascular disease. In light of recent trials, namely the CANTOS study, showing the enormous potential of anti-inflammatory therapies and in particular those targeted to IL-1β, a change in therapeutical management of cardiovascular diseases is coming about. The NLRP3 inflammasome is a multiprotein complex that assembles to engage the innate immune defense by processing the maturation of pro-inflammatory cytokines IL-1β and IL-18. Substantial evidence has positioned the NLRP3 inflammasome at the center of vascular disease progression, with a particular significance in the context of aging and the low-grade chronic inflammation associated (inflammaging). Therefore, pharmacological blockade of the NLRP3 inflammasome and its end products has arisen as an extremely promising tool to battle vascular disease. In this review, we discuss the mechanisms by which the NLRP3 inflammasome contributes to vascular disease, with particular attention to the consequences of aging, and we enumerate the therapeutic options available to combat this recurrent villain.

## 1. Introduction

Inflammasomes are a group of cytosolic multiprotein complexes that assemble to engage the innate immune defense by processing the maturation of pro-inflammatory cytokines [1]. Among them, the most studied is the NLRP3 inflammasome. A meaningful role of the NLRP3 inflammasome in age-associated diseases has been evidenced [2], which has paved the way for novel pharmacological interventions to ablate the inflammasome’s effects in neurodegeneration and vascular disease, among others. Multiple mechanisms related to aging and comorbidities such as obesity or diabetes can alter the complex process governing NLRP3 activation, leading to a chronic hyperinflammatory state [3]. In fact, NLRP3 inflammasome over-activation and the subsequent increase in inflammatory cytokines, especially IL-1β, has been frequently linked to atherosclerosis, diabetes, and related chronic sequels [4,5,6].

For all these reasons, understanding the mechanisms regulating NLRP3 inflammasome activation and subsequent signaling is vital to gain insight into potential therapeutic strategies to prevent NLRP3 inflammasome-driven diseases. Furthermore, targeting the NLRP3 inflammasome can offer therapeutic benefits in chronic vascular diseases and subsequent sequels, particularly in light of clinical trials such as CANTOS, which have demonstrated that the inhibition of one of the main products of NLRP3 inflammasome activation, IL-1β, can diminish the risk of major cardiovascular events [7].

## 2. NLRP3 Inflammasome: Mechanisms and Physiological Function

Priming, assembly and activation of the NLRP3 inflammasome can be triggered by a plethora of pathogenic products and endogenous danger signals sensed by pattern recognition receptors (PRRs) [8]. In a broad context, an array of germline-encoded PRRs mediate the immune surveillance, which can be membrane-bound, such as Toll-like receptors (TLRs) [9]. The NLRP3 inflammasome comprises a sensor protein that belongs to a further set of intracellular PRRs known as the nucleotide-binding oligomerization domain-like receptors (NLRs), including 22 members in humans [8]. NLRP3 receptor is unique among innate immunity elements as it can recognize a vast variety of pathogenic and non-pathogenic endogenous stressors, unlike the majority of PRRs, which have limited specificity to one or few unrelated stimuli [10]. As the sources of NLRP3 inflammasome activation are diverse, this complex could also mediate a heightened state of sterile inflammation when over-activated. Indeed, the NLRP3 inflammasome has been implicated in the pathogenesis of various autoimmune, autoinflammatory, and chronic inflammatory diseases [11].

The NLRP3 inflammasome trimeric complex encompasses several distinct protein/protein interaction domains and acts as a platform to activate the effector protein caspase-1 that prompts the maturation of pro-inflammatory cytokines interleukin (IL)-1β and IL-18, as well as pyroptosis [12]. The complex also includes an adaptor protein with two protein interaction domains, the carboxy-terminal caspase-recruitment domain (CARD) and amino-terminal pyrin domain (PYD), known as the apoptosis-associated speck-like protein containing a CARD (ASC) that bridges the NLRP3 receptor to the procaspase-1 [10]. The NLRP3 protein possesses a PYD domain; a central nucleotide-binding and oligomerization (NACHT) domain that is crucial for the inflammasome assembly and function owing to its ATP-binding properties; and leucine-rich repeats (LRRs), which are thought to be involved in autoinhibition via folding back onto the NACHT domain [10,12,13]. Recent studies also suggest the importance of LRRs and NACHT interaction with the NIMA-related kinase 7 (NEK7) for the activation of the NLRP3 inflammasome [14,15].

**Figure 1:**
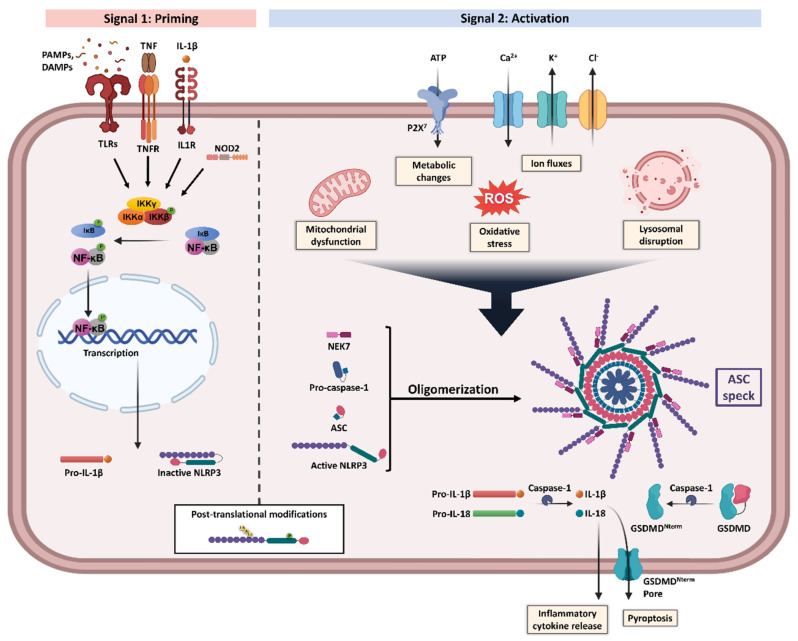
**NLRP3 inflammasome priming and activation**. NLRP3 inflammasome activation requires two steps, Signal 1 (priming, **left**) and Signal 2 (assembly and activation, **right**). Priming is initiated by various exogenous and endogenous stressors that engage pattern recognition receptors (PRRs) such as Toll-like receptors (TLRs) and nucleotide-binding oligomerization domain-containing protein 2 (NOD2), or cytokines receptors like interleukin-1 receptor type 1 (IL-1R1) and tumour necrosis factor receptor (TNFR), leading to nuclear factor-κB (NF-κB) activation and gene transcription of NLRP3 and proIL-1β. Multiple post-translational modifications including ubiquitylation and phosphorylation are crucial for the licensing of the NLRP3 protein activation. Signal 2 is induced by numerous triggers including mitochondrial dysfunction, metabolic changes, oxidative stress, ion fluxes, and lysosomal disruption. The formation of NLRP3 inflammasome protein complex activates caspase-1 that claves pro-IL-1β and pro-IL-18 into their bioactive inflammatory forms. Gasdermin D (GSDMD) is also cleaved and the resulting GSDMD amino terminal (GSDMD^Nterm^) binds to the membrane, forming pores and inducing pyroptosis. Apoptosis-associated speck-like protein containing a CARD (ASC), damaged-associated molecular patterns (DAMPs), IκB kinase (IKK), inhibitor of NF-κB (IκB), NIMA-related kinase 7 (NEK7), P2X purinoceptor 7 (P2X^7^), pathogen-associated molecular patterns (PAMPs), reactive oxygen species (ROS), and tumour necrosis factor (TNF). Created with BioRender.com. accessed date (15 December 2021).

Furthermore, the activation of the NLRP3 inflammasome is tightly regulated through a two-step process (Figure 1): (1) priming and (2) assembly and activation [10]. The priming signal initiates a sequence of events to upregulate the insufficient levels of NLRP3 components existing in the cells at the resting state [16]. This transcriptional upregulation can be induced by diverse damaged-associated molecular patterns (DAMPs) and pathogen-associated molecular patterns (PAMPs) through various PRRs including TLRs, NLRs, and cytokines’ receptors, which result in nuclear factor-κB (NF-κB) activation and gene transcription of NLRP3 as well as main pro-inflammatory genes, such as pro-IL-1β [16]. During this step, NLRP3 is still controlled through a combination of post-translational modifications (PTMs), which have been identified to be crucial in regulating NLRP3 inflammasome activation [10]. Of these modifications, phosphorylation can induce or inhibit activation depending on the site and stage of NLRP3 activation [17]. Furthermore, ubiquitination and deubiquitination have an essential role in the degradation and activation of NLRP3 [16]. Accordingly, NLRP3 remains in an auto-suppressed inactive state, prepared to receive the activation signal [10,16].

Multiple cellular signals trigger the activation and assembly of NLRP3 inflammasomes including ion fluxes such as K^+^ efflux and Ca^2+^ influx, lysosomal disruption, metabolic changes, mitochondrial dysfunction, and ROS production [6]. However, the roadmap of NLRP3 inflammasome activation is extremely intricated, as many pathways intersect and are interrelated [12]. Once NLRP3 senses the activation signal, it begins the recruitment and oligomerization of ASC and pro-caspase-1 through homophilic interactions to form large speck-like structures [6]. Pro-caspase-1 recruitment enables proximity-induced self-cleavage and activation, producing catalytically active species of caspase-1 [18]. Subsequently, a proteolytic cleavage by caspase-1 generates the bioactive forms of the inflammatory cytokines IL-1β and IL-18 and mediates the activation of the cytosolic protein gasdermin D (GSDMD) [6]. The GSDMD-N domain can bind to membrane lipids and form pores that could mediate the non-conventional secretion of IL-1β and IL-18 and trigger pyroptosis, an inflammatory type of programmed cell death [6,18,19].

## 3. NLRP3 Inflammasome in Inflammation and Vascular Disease

Inflammation is a major cause underlying vascular diseases. Acute inflammation is triggered by acute events that provoke a systemic response in order to restore homeostasis. However, in the case of chronic inflammation, chronically inflamed tissues produce a prolonged response that induces the migration of leukocytes to the tissues, ultimately producing tissue degeneration and organ damage [20]. With aging, this persistent inflammatory state leads to low-grade sterile chronic inflammation, so-called “inflammaging” [21]. Inflammaging is to date considered a major driver of age-associated diseases and, in particular, of vascular disease.

Both end products of the activation of NLRP3, the cytokines IL-1β and IL-18, are pro-inflammatory and have been associated with several acute and chronic inflammatory diseases, including cardiovascular diseases [22]. IL-1β, among other cytokines and factors, is able to activate the canonical NF-κB signalling pathway [23]. NF-κB is a redox-sensitive transcription factor that, in physiological conditions, is inhibited in the cytoplasm by its binding to IκB. Upon activation, the inhibitor is phosphorylated by the IκB kinase complex and degraded by the proteasome. These events result in the translocation of the subunits p50 and p65 to the nucleus, which activates a variety of genes that are involved in inflammation, among others [24]. In fact, some of the genes regulated by NF-κB are actually pro-IL-1β and pro-IL-18 genes, which code for the precursor proteins cleaved by the inflammasome [25]. Hence, NF-κB is a key modulator of the NLRP3 inflammasome by inducing the priming phase. Besides, IL-1β also induces inflammation by the activation of JNK and p38 [23]. JNK and p38 are mitogen-activated protein kinases (MAPKs), which are phosphorylated and activated by a cascade of MAPK after the binding of IL-1β to its receptor [23]. Once activated, JNK and p38 induce the activation of transcription factors that are involved in the expression of pro-inflammatory genes, such as ATF2 and AP1 [23]. Another consequence of NLRP3 activation is the induction of pyroptosis, a pro-inflammatory cell death leading to an increment in the production and release of IL-1β and IL-18, which contributes to the maintenance of a pro-inflammatory environment [26] and has been demonstrated to contribute to vascular disease [27].

Atherosclerosis is the main cause underlying vascular disease [28] and there are several pieces of evidence pointing out that inflammation and NLRP3 play an important role in this disease [29]. Oxidized LDL (oxLDL), which is known to accumulate in the vessel wall during atherosclerosis and is phagocyted by macrophages, activates NLRP3 by processes related to influx of Ca^2+^, reactive oxygen species, and mitochondrial dysfunction, among others [27]. Moreover, it is known that cholesterol crystals, which are also present in atherosclerotic lesions, can be phagocyted by human macrophages and induce the production and secretion of IL-1β by the activation of NLRP3 inflammasome, triggering inflammation [30]. There is also a study in which the injection of cholesterol crystals to control mice induced inflammation, while it had no inflammatory effects in mice deficient in NLRP3 components, assessing the important role of NLRP3 components in inflammatory processes in vivo [31].

In two models of atherosclerosis, ApoE^−/−^ mice with high-fat diet and ApoE^−/−^ mice with high-fat and high-methionine diet, the levels of IL-1β and IL-18, macrophage infiltration and atherosclerotic lesions were increased compared with control mice, while the silencing of NLRP3 gene reduced all of these effects [32]. Moreover, NLRP3 inflammasome proteins and its produced cytokines have been shown to be over-expressed in human carotid atherosclerotic plaques, especially in unstable plaques [5]. Over-activated NLRP3 inflammasome was also related to atherosclerosis in diabetic patients, and NLRP3 knockdown reduced and stabilized atherosclerotic plaque in a diabetic atherosclerosis mouse model [33].

IL-1β is known to be produced by hematopoietic cells, as well as vascular endothelial and smooth muscle cells under inflammatory conditions, in which they also induce proliferation and have inflammatory effects contributing to the process of atherosclerosis [5]. Moreover, IL-1β is known to provoke the expression of adhesion molecules in vascular endothelial cells, which results in the migration of monocytes and other leukocytes [34]. Moreover, the other end product of NLRP3, IL-18, also contributes to atherogenesis. The arterial tissue from patients with atherosclerosis showed an increased expression of IL-18 and its receptor compared with healthy tissue [35]. Hence, it has been demonstrated that mononuclear macrophages present in atherosclerotic lesions express IL-18, and endothelial and vascular smooth muscle cells (VSMCs) as well as mononuclear macrophages express the IL-18 receptor [35]. Furthermore, the deficiency of IL-18 in a mice model of atherosclerosis showed decreased atherosclerotic lesions [36]. All of these findings reinforce the significant role of NLRP3 and its derived cytokines in the pathogenesis of atherosclerosis.

The detrimental role of NLRP3 inflammasome and IL-1β from VSMCs in atherosclerosis has recently been challenged by a study from Gomez et al., showing some atheroprotective effects of IL-1β [37]. This study shows smaller lesions from SMC-specific IL1r1^−/−^ Apoe^−/−^ mice, but also reports signs of instability in these animals and SMC-lineage tracing Apoe^−/−^ mice with advanced atherosclerosis treated with anti-IL-1β. A considerable number of studies have reported that NLRP3 inflammasome governs the pro-inflammatory phenotypic switch of VSMCs during atherogenesis [38,39], and contributes to vascular inflammation [40,41], possibly favouring plaque instability and disease progression. Besides, the CANTOS study has provided a great body of evidence on the cardiovascular benefits of anti-IL-1β therapy. Anyhow, this trial also showed inhibition of beneficial outward remodelling on treated patients, and this, together with the work from Gomez et al. [37], highlights the potential adverse effect of excessive IL-1β inhibition within VSMCs. It is important to note that the results by Gomez et al. [37] were obtained under extreme inhibitory conditions, such as the IL1r1 knockout mice, and these might not reflect the reduction circumstances from a human drug treatment.

From a clinical point of view, NLRP3 inflammasome activation has been suggested as a relevant indicator of cardiovascular disease severity and quantification of its components and end products in circulating cells and plasma/serum arise as novel potential biomarkers. Regarding coronary atherosclerosis, NLRP3 is thought to be correlated with the severity of acute coronary syndrome (ACS); that is patients with ACS present higher levels of NLRP3 protein in peripheral blood monocytes and higher levels of IL-1β and IL-18 in plasma than control patients, and the levels of NLRP3 increased with the severity of the disease [42]. Another study demonstrates that the levels of caspase-1 are higher in aortas from patients with coronary disease compared with healthy patients, and that those levels also correlate with the severity of the disease [43]. Moreover, in both studies, it has been observed that patients with typical cardiovascular disease risk factors present higher levels of NLRP3 inflammasome activation [42,43].

NLRP3 has not only been linked to atherosclerosis, but also to other harmful vascular processes, such as abdominal aortic aneurysm (AAA). There is a study in which the expression of NLRP3 was analysed by means of ASC and caspase-1 protein levels from biopsies of patients suffering AAA, reporting increased levels of expression compared with healthy patients [44].

In the context of diabetes, hyperglycemia would lead to mitochondrial dysfunction and oxidative stress, as well as chronic inflammation, all of which shall induce the NLRP3 inflammasome [45]. In fact, in drug-naïve type 2 diabetic patients, an increase in the expression of inflammasome components NLRP3 and ASC was observed in monocyte-derived macrophages, as well as higher serum levels of IL-1β and IL-18 [46]. In line with these results, a diabetic rat model showed excessive activation of NLRP3 inflammasome accompanied by cardiac inflammation, pyroptosis, and fibrosis, which were suppressed by NLRP3 gene silencing therapy [47].

Although IL-1β has been more extensively studied, IL-18 and a cytokine released downstream of both end products of NLRP3 inflammasome, IL-6, both play a crucial role in vascular pathogenesis, and pharmacological interventions targeting these two interleukins are emerging.

## 4. NLRP3 Inflammasome and Vascular Cell Senescence

Vascular aging is a complex and multifaceted process that provokes complex structural, molecular, and functional changes in the vasculature. In blood vessels, aging is a major risk factor for developing vascular diseases, such as hypertension or atherosclerosis, as well as related cardiovascular events. Vascular cell senescence is a main hallmark of vascular aging. In response to a large array of stressors, vascular cells may undergo a series of functional and morphological changes that ultimately lead to growth arrest and the acquisition of a pro-inflammatory senescence-associated secretory phenotype (SASP) [48]. Cell senescence is a main driver of the pathogenesis of aging-associated diseases, and senescent cells from all vascular cell types have been shown to contribute to disease progression [49]. In humans, the accumulation of vascular senescent cells has been well characterized in human atherosclerotic lesions and seems to be critical for the development and progression of the lesion [50,51].

The link of vascular cell senescence and NLRP3 inflammasome activation has only recently been discovered in endothelial cells [52,53], but is likely that this mechanism is replicated by other vascular cell types. The endothelial senescence phenotype is characterized by typical changes of a dysfunctional endothelium, like impaired reactivity with defective relaxation, enhanced pro-inflammatory and pro-coagulant status, remodelling, and atherogenesis, among others [48]. In cultured human endothelial cells, the pro-inflammatory cytokine IL-1β promotes DNA damage and cell senescence [54]. It also favours the expression of NLRP3 and pro-IL-1β proteins, as well as the activation of the NLRP3 inflammasome and the release of additional IL-1β in an auto-inflammatory loop [53]. Inversely, the pharmacological NLRP3 assembly blocker MCC950 averts the endothelial cell senescence triggered by IL-1β [53]. Besides, the in vivo infusion of IL-1β to mice results in enhanced vascular NLRP3 expression, endothelial dysfunction, and a loss of vasodilatory capacity, which can also be prevented by MCC950 [53]. Similarly to MCC950, Ang-(1-7), the protective heptapeptide of the renin-angiotensin system, and the anti-aging protein klotho can equally inhibit the endothelial priming and activation of the NLRP3 inflammasome induced by IL-1β, thus preventing endothelial cell senescence in vitro and endothelial dysfunction in vivo [53].

Not only drugs, but also nutrients may modulate endothelial cells’ senescence driven by the NLRP3 inflammasome. Hence, potato-derived flavonoids have been described to prevent NLRP3 inflammasome activation, probably as a consequence of decreased ROS formation, and to attenuate premature endothelial cell senescence in an in vivo model of diabetes induced by D-galactose [55]. Besides IL-1β, the NLRP3 inflammasome has been shown to mediate endothelial cell senescence elicited by other stimuli such as oxidative stress induced by hydrogen peroxide [56].

Although additional research is needed in this field, the NLRP3 inflammasome arises as a promising target to prevent vascular cell senescence, thus delaying premature vascular aging and its deleterious consequences.

## 5. Oxidative Stress and the NLRP3 Inflammasome

**Figure 2:**
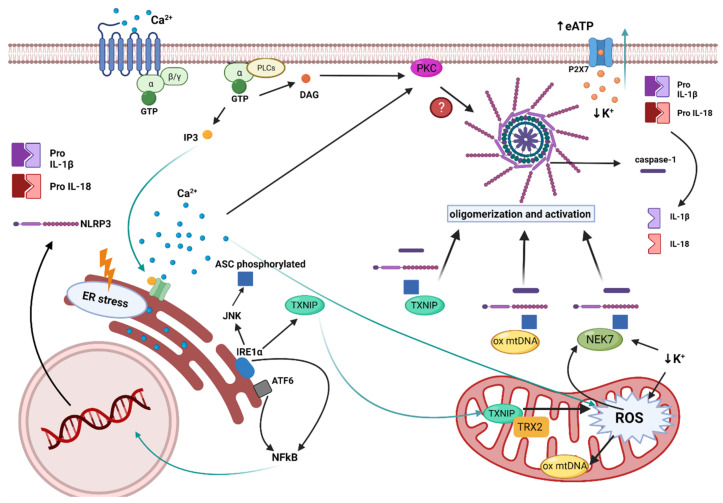
**Role of ER stress and mitochondrial dysfunction in NLRP3 activation**. (1) Different stimuli induce ER stress, which produce the activation of IRE1α and ATF6. These proteins activate NFkB, which is translocated to the nucleus and induces the transcription of the genes coding NLRP3, pro-IL-18, and pro-IL-1β. Moreover, IRE1α induces the phosphorylation of ASC, needed for the formation and activation of the inflammasome, and the activation of TXNIP, involved in the oligomerization and activation of the inflammasome. (2) Accumulation of ROS in the mitochondria, owing to different factors such as the reduction of intracellular K^+^ or the increment of Ca^2+^ inside the mitochondria, causes mitochondrial dysfunction. This mitochondrial dysfunction results in the production of oxidized mitochondrial DNA and activation of NEK7, which induce the oligomerization and activation of the inflammasome. As a result of the oligomerization and activation of NLRP3, the enzyme caspase-1 cleaves the inactive forms of IL-1β and IL-18, resulting in the active forms of both cytokines. Created with BioRender.com. accessed date (15 December 2021).

Excessive oxidative stress plays a crucial role in inflammation, especially in chronic states. Aging is the most influential cause underlying vascular dysfunction and, in this setting, the interplay of persistent oxidative stress and sterile inflammation (inflammaging) arises as a major determinant of the pathogenesis [21]. Activation of the NLRP3 inflammasome in response to endogenous oxidative stress is clearly at the center of this process (Figure 2), with three main DAMPs causing this effect: reactive oxygen species (ROS), Ca^2+^ mobilization, and K^+^ efflux [45]. In fact, K^+^ efflux seems to be a recurrent feature governing NLRP3 inflammasome activation by oxidative stress and inflammatory stimuli [57]. Besides, membrane permeation, lysosomal damage, mitochondrial dysfunction, and ROS production are interrelated cellular events that can mutually cause each other [58]. All of these can be initially induced by increased extracellular ATP (eATP) released by inflammatory, senescent, apoptotic, and necrotic cells [59]. As a result of vascular aging, cells within the vessel wall are more prone to these degenerative processes [21], thus eATP will be increased. Furthermore, a metabolic imbalance associated with high-calorie intake, obesity, and diabetes also results in greater levels of eATP, and enhanced purinergic signaling is thus pivotal in the metabolic syndrome [60]. Hence, vascular aging and the comorbidities associated with the metabolic syndrome will ultimately result in enhanced NLRP3 activation, with a chain of actions involving eATP, mitochondrial damage, ROS, and ultimately K^+^ efflux. Downstream of K^+^ efflux, NEK7 has been demonstrated to be indispensable for the oligomerization and activation of the NLRP3 inflammasome [14], with mitochondrial ROS and Ca^2+^ mobilization as part of the mechanism [61]. Besides, the expression of NEK7 and the NLRP3 inflammasome was found to be significantly increased in arteries from patients with diabetic foot, with vascular smooth muscle cells showing the greater expression [62].

Oxidative stress is deleterious when disproportionate and can be counterbalanced by antioxidants. As a result of mitochondrial dysfunction, not only ROS augment, but also antioxidant levels decrease within the cells, impairing the balance [45]. This results in overproduction of ROS such as superoxide, which, in the endothelium, has been shown to inhibit eNOS and induce the expression of adhesion molecules, leading to endothelial dysfunction [6]. Besides, mitochondrial dysfunction can also lead to an increased NAD^+^/NADH ratio, another source of oxidative stress contributing to vascular disease [63]. Sirtuins, a family of proteins that can sense alterations in the NAD^+^/NADH ratio and respond by reprogramming immune, metabolic, and bioenergetic pathways, have been shown to decrease with chronic inflammation [45]. Therefore, in the vascular milieu of low-grade sterile inflammation, the defense mechanism of sirtuins will be impaired. In particular, sirtuin 2 has been shown to protect against oxidative stress-induced vascular injury by deacetylation and ubiquitination of the oxidative stress injury-related protein poly(ADP-ribose) polymerase 1 (PARP1) in mice [64]. Besides, one of the mechanisms by which sirtuins mitigate chronic inflammation in the vasculature is the inhibition of the NLRP3 inflammasome [65].

On the other hand, endoplasmic reticulum (ER)stress can also lead to the activation of the NLRP3 inflammasome through ROS and release of Ca^2+^ [66]. Different stimuli induce ER stress, which produces the activation of the sensors: inositol-requiring enzyme 1α (IRE1α) and activating transcription factor 6 (ATF6) [57]. These proteins activate the unfolded protein response (UPR), in order to combat ER stress. When UPR fails to restore ER homeostasis, maladaptive UPR induces risk factors for cardiovascular disease, including increased ROS production, inflammation, and apoptosis, which further aggravate cardiovascular disease [67]. In particular, these proteins activate NFkB, which is translocated to the nucleus to induce the transcription of the genes coding NLRP3, pro IL-18, and pro IL-1β, thus activating the inflammasome´s priming [6]. Furthermore, IRE1α induces the phosphorylation of ASC, needed for the assembly and activation of the NLRP3 inflammasome [57].

As discussed earlier, oxidative stress can trigger NLRP3 inflammasome activation. Besides, IL-1β released by the NLRP3 inflammasome has been shown to increase oxidative stress, mainly by inducing NADPH oxidase [68], a crucial producer of ROS within the vascular system. Hence, there is a positive loop that links oxidative stress and NLRP3 inflammasome activation, which plays a pivotal role in vascular aging and the associated chronic inflammatory state. Within the atherosclerotic plaque, this vicious cycle of oxidative stress and NLRP3 interplay has been shown to play a crucial role, with monocyte/macrophage/foam cells, endothelial cells, and VSMCs taking part in this mechanism during atherogenesis [69]. As outlined in Section 3, accumulated oxLDL within the vessel wall is phagocyted by macrophages, activating NLRP3 by processes related to influx of Ca^2+^, reactive oxygen species, and mitochondrial dysfunction [27]. Besides, NLRP3-mediated pyroptosis has been reported in endothelial cells, as a response to elevated ROS induced by hyperhomocysteinemia [70], and this process shall be a hallmark of endothelial dysfunction within the atherosclerotic plaque [71]. With regards to VSMCs, NLRP3 inflammasome causes migration and proliferation of these cells by means of angiotensin II-induced ROS, as evidenced in vitro and in vivo [72].

## 6. Pharmacological Interventions to Mitigate NLRP3 Inflammasome Activation in Vascular Disease

**Figure 3:**
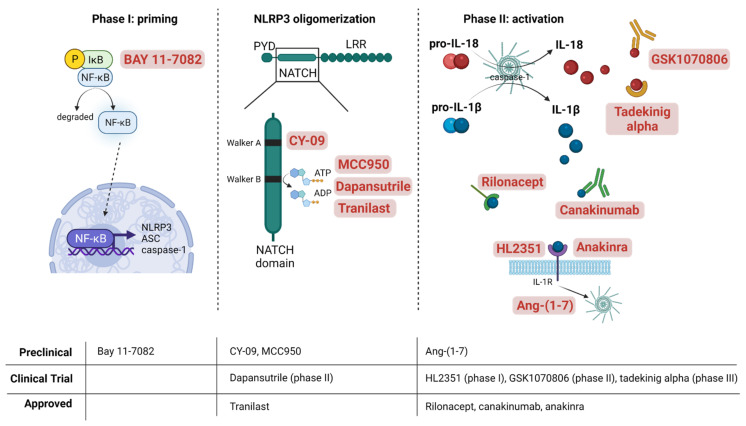
**Pharmacological interventions against NLRP3 inflammasome**. Graphical representation depicting the simplified mechanism of action of the most representative investigated drugs that target the NLRP3 inflammasome machinery. The drugs were classified into three groups depending upon which of the phases of the chronological pathway for NLRP3 inflammasome activation they exert their inhibitory action, namely, phase I or priming, NLRP3 protein oligomerization, and phase II or activation. At the bottom of the image, a table summarizes the current state of development of the different molecules and drugs, according to the literature. Created with BioRender.com. accessed date (20 January 2022).

The interest in targeting the NLRP3 inflammasome arises as its anomalous activity or disrupted genetic expression directly link with the development of many chronic inflammatory diseases. Some pharmacological interventions comprise drugs that target NLRP3 inflammasome as a whole, mitigating its activity, whereas others directly interact with the components and products of the inflammasome to hamper its oligomerization and impede their direct effects (Figure 3). Besides, another interesting pharmacological strategy consists of reducing oxidative stress by the use of antioxidants to indirectly reduce NLRP3 inflammasome activation and the consequent chronic inflammatory state.

On one hand, some compounds act as indirect inhibitors of the priming phase in NLRP3 inflammasome activation. As mentioned above, redox signaling is a main driver of NLRP3 inflammasome activation. In this regard, the clinical use of drugs with antioxidant properties constitutes a promising strategy to alleviate chronic activation of the NLRP3 inflammasome. For example, resveratrol, by modulating sirtuin 1 activity, was able to inhibit NLRP3 activation and protected against vascular injury in vivo [73]. One of the mechanisms of resveratrol is the uncoupling of eNOS and the inhibition of NADPH oxidase activity at the endothelial level [74], which might hold great benefit against NLRP3 overactivation. TXNIP, which acts as a sensor of ROS and mediator of ER stress leading to NLRP3 inflammasome activation, was shown to be essential in the mechanism of NLRP3-mediated endothelial senescence [52], and its genetic ablation has been demonstrated to delay the progression of the atherosclerotic lesion in mice [75]. Recently, a new compound that specifically inhibits TXNIP expression, called SRI-37330, has been developed. This drug was able to improve glucose homeostasis in diabetic mice [76], although its pharmacological application at the moment is mostly oriented to the management of diabetes rather than the NLRP3-mediated over-inflammation. The antioxidant hormone melatonin has also been found to downregulate the TXNIP/NLRP3 pathway in LPS-induced endometritis [77]. In another study, melatonin prevented atherosclerosis in a mice model of obesity by blocking NLRP3 inflammasome activation by means of SIRT-1 dependent deacetylation and inhibition of NF-kB [78]. The bidirectional connection between ROS and NF-kB activation is well known, thus blockade of ROS/NF-kB signaling has been also targeted. For example, Bay 11-7082 antagonizes IKKβ activity by inhibiting its phosphorylation, which prevents nuclear translocation and activation of NF-κB [79]. This compound was found to improve the inflammatory profile, including a reduction in IL-1β production, of an experimental model of psoriasis-like dermatitis [80]. Hydrogen sulfide (H_2_S) has also been shown to reduce oxidative stress and subsequently diminish NLRP3 inflammasome activation in endothelial cells [81], as well as to mitigate deleterious vascular remodeling in aortic aneurysm [82].

The strategy in the design of small molecules for the blockade of NLRP3 relies on impeding NLRP3 self-oligomerization or heterologous assembly with ASC by inhibition of its ATPase activity. The NATCH domain within the NLRP3 structure is a major target for small-molecule compounds inhibiting NLRP3 inflammasome activation, namely, MCC950, dapansutrile (OLT1177), CY-09, or tranilast, among others. MCC950 is considered the most potent and selective inhibitor of NLRP3 inflammasome, able to block canonical and non-canonical NLRP3 activation [83]. Recently, it was determined that MCC950 modifies the active conformation of NLRP3 into a closed structure that impedes its oligomerization [84]. To do this, MCC950 binds to a specific residue within the walker B motif in the NATCH domain of NLRP3 [83]. Corcoran et al. have extensively reviewed the actions of MCC950 in more than 100 preclinical models of inflammatory diseases [85]. MCC950 has demonstrated antioxidant properties by inducing the tissular overexpression of sirtuin 1 and superoxide dismutase (SOD) in treated mice [86]. Promising preclinical results have been obtained in neurology, as MCC950 decreased chronic neuroinflammation and cognitive impairment in animal models of neurodegeneration [87,88]. Likewise, in the brain of senescence-accelerated mouse prone 8 (SAMP8) and wild type aged mice, MCC950 reduced neuron death, also improving cognitive impairment [89,90]. In a vascular setting, this drug has demonstrated atheroprotective activity by reducing the size of the atherosclerotic plaque [91] and protection against myocardial infarction in a pig model [92]. In db/db mice, MCC950 treatment reversed the impaired endothelial dysfunction [93], while in mice mesenteric microvessels and human cells, MCC950 prevented the detrimental effects induced by several pro-inflamatory adipokines [53,94].

In spite of the promising results in animal models, MCC950 is not involved in any ongoing clinical trial. By contrast, dapansutrile (also known as OLT1177) has been evaluated in a phase I clinical trial, where its oral administration demonstrated safety and was well tolerated in healthy humans [95]. Moreover, in patients with gout flare, 8-day OLT1177 treatment reduced joint pain and plasma IL-6 levels [96]. OLT1177 inhibits NLRP3-ASC and NLRP3-caspase-1 interaction, and thus NLRP3 oligomerization, in neutrophils and human monocyte-derived macrophages [95]. A current clinical trial will evaluate its safety and pharmacodynamics in subjects with stable systolic heart failure with signs of systemic inflammation (NCT03534297) [97].

Tranilast is an analogue of a tryptophan metabolite, an anti-allergic drug used in the treatment of bronchial asthma and several dermatitis-related alterations [98]. Even though tranilast showed an in vivo activity around 5–10 times less potent than MCC950 [99], it prevented acute inflammation and tissue damage in a monosodium urate crystals (MSU)-dependent arthritis mouse model, as well as metabolic disorders in high-fat diet (HDF)-induced diabetic mice [99]. Moreover, aside from its anti-inflammatory and anti-thrombotic effects, tranilast exerted antioxidant actions. It has been demonstrated to scavenge ROS and directly inhibit xhantine oxidase activity in vitro [100], as well as to reduce TXNIP expression and ROS production in streptozotocin-treated rats [101]. In other in vivo settings of arthritis, cryopyrin-associated periodic syndrome (CAPS), and type 2 diabetes, tranilast prevented NLRP3-dependent inflammatory effects by directly binding to the NATCH domain of NLRP3 [99]. Similarly, the glitazone derivate CY-09 has demonstrated preventive and therapeutic potential in mice models of gout, type 2 diabetes, and CAPS [102]. Among the current small drugs, CY-09 is the only one directly interacting with NLRP3 walker A motif in the NATCH domain to exclude the ATP association to NLRP3 [102].

None of the medications targeting the NLRP3 protein complex are available yet in the clinic. The repurposing of several approved drugs that have been demonstrated to directly inhibit the complex appears as a promising strategy to tackle NLRP3 inflammasome over-activation. In this regard, new pharmacological interventions against IL-1β in NLRP3-driven diseases are the only clinical available treatments, including the recombinant IL-1 receptor antagonist anakinra, the neutralizing IL-1β humanized antibody canakinumab (also known as ACZ885), and the soluble decoy IL-1β receptor rilonacept [103]. The three of them have demonstrated efficacy in the long-term treatment of Muckle–Wells syndrome, familiar Mediterranean fever, and other CAPS [104,105]. Moreover, these drugs are employed in other inflammatory conditions. While anakinra was the first FDA-approved drug for rheumatoid arthritis [106], a phase III clinical trial has demonstrated the efficacy of rilonacept in immune recurrent pericarditis treatment [107]. In addition, the CANTOS trial showed the capability of canakinumab to reduce inflammation in the pathogenesis of atherosclerosis independently of other traditional risk factors [7]. Apart from the direct blockade of IL-1β or its receptor, these drugs have demonstrated to exert an antioxidant effect. While anakinra was able to modulate mitochondrial ROS production by activating SOD2 in mice [108], a clinical study concluded that rilonacept reduced the activation of endothelial cell NADPH oxidase in patients with chronic kidney disease [109]. This is consistent with the described pathological mechanism of IL-1β in vascular cells, by which the cytokine has proven to induce NADPH oxidase [68], a key source of ROS in vascular tissues. Besides, inhibition of IL-1β signaling has been shown to reduce NADPH oxidase activation in diabetic rats [110].

Lately, improved biological drugs have been developed. HL2351 is a recombinant protein consisting of an antibody fragment fused to two human IL-1b receptor antagonist components. Although it shares mechanism of action with anakinra, HL2351 comprises a longer circulation half-life and might reduce dose frequency and improve therapeutic efficacy [111]. Although a clinical trial was designed aiming to test its efficacy and safety and to evaluate pharmacokinetics in CAPS patients, it had to be terminated owing to difficulties in recruitment (NCT02853084). Nevertheless, inhibition of IL-1β function might have a greater immunosuppressive effect than inhibition of NLRP3 itself, as this cytokine can also be released by other inflammasomes. In this sense, there is interest in the identification of other small drugs and compounds that indirectly oppose IL-1β effects. The heptapeptide Angiotensin (Ang)-(1-7) is the physiological antagonist of Ang II in the renin-angiotensin-aldosterone system (RAAS), opposing Ang II-induced detrimental vascular effects through Mas receptors [112]. Ang-(1-7) was found to exert anti-inflammatory and anti-senescent effects over RAAS-dependent and independent pro-inflammatory stimuli, including IL-1β [54,113]. The action of Ang-(1-7) against IL-1β might be in part mediated by its antioxidative capability. Ang-(1-7) is able to inhibit IL-1β-induced iNOS expression, NADPH oxidase activity, and subsequent NF-κB activation in vascular smooth muscle cells [113]. Moreover, in vitro and in vivo, Ang-(1-7) is able to blunt NLRP3 inflammasome/IL-1β over-activation loop [54]. Furthermore, Ang-(1-7) action stimulates cytoprotective mediators such as the Nrf2/heme oxygenase (HO-1) pathway [54]. It should be noted that several Nrf2-activating compounds such as sulforaphane can suppress IL-1β secretion by upregulating HO-1 and maintaining the inactive conformation of TXNIP [114]. However, as a compensatory mechanism, some reports have also observed that NLRP3 inflammasome activators such as cholesterol can induce the expression of Nrf2-dependent genes, concluding that Nrf2 may be also necessary in NLRP3 activation by triggering the assembly of ASC specks [115].

In contrast, as the NLRP3 inflammasome also releases IL-18, specific inhibition of IL-1β does not mitigate IL-18-driven inflammation. The subsequent release of IL-6 by target cells [5], induced by both IL-1β and IL-18, will nonetheless be reduced, but not completely. This has been evidenced in the CANTOS population, where residual IL-18 and IL-6 inflammation after IL-1β inhibition correlated with greater cardiovascular risk [116]. Therefore, on the one hand, targeting IL-1β can reduce inflammation triggered by all kinds of inflammasomes, but the effects of IL-18 will not be counteracted. When directly targeting the NLRP3 inflammasome, a complete inhibition of the inflammatory signals triggered by IL-1β and IL-18 will be accomplished, but other inflammasomes such as AIM2 could contribute to both cytokines´ processing and release [8].

CAPS are associated with a gain-of-function missense mutation in exon 3 of the NLRP3 gene. Current clinical trials evaluating pharmacological interventions to mitigate NLRP3 inflammasome activation have been designed in CAPS patients to counteract this gain-of-function (tranilast NCT03923140, canakinumab NCT01576367, or rilonacept NCT00288704), rather than its malfunction, leading to autoimmune or inflammatory diseases. This unique focus on CAPS disease of the clinical trials with NLRP3 inhibitors is one of the reasons that MCC950, acknowledged as the most potent NLRP3 inflammasome inhibitor according to the preclinical results, has not been continued into clinics, as binding of MCC950 to NLRP3 has been shown to be impeded by CAPS-associated mutation [117]. Moreover, MCC950 has shown poor pharmacokinetic and toxicokinetic properties [86]. By contrast, next-generation inhibitors based on the beneficial properties of MCC950 are being developed and hold great promise [118].

A dark side of persistent systemic inhibition of the NLRP3 inflammasome linked to its adverse effects must be acknowledged and should guide prescription of the aforementioned drugs. In particular, dampening the innate immune response can lead to increased infection risk, as evidenced in the CANTOS trial, where canakinumab treated patients showed higher rates of neutropenia and significantly more deaths attributed to infection or sepsis, although these persisted at a very low incidence (incidence rate: 0.31 vs. 0.18 events per 100 person-years) [7]. On the other hand, inhibition of IL-1β resulted in a lower incidence of arthritis, gout, and osteoarthritis than the placebo group [7], showing a global benefit in the alleviation of aging-associated diseases. One promising strategy to reduce systemic side effects that deserves further research is aiming local delivery of drugs targeting the NLRP3 inflammasome to the vascular tissue. This could be attempted using vehicles such as nanoparticles [119], liposomes [120], or extracellular vesicles [121], decorated with either aptamers or nanobodies specific to the endothelium.

Most approved pharmacological interventions have targeted IL-1β and many have targeted the NLRP3 inflammasome, but the potential clinical utility of inhibiting IL-18 and the downstream cytokine IL-6, in order to combat vascular disease, although less explored, also holds great promise. Drugs such as human IL-18 binding protein, tadekinig alfa [122], or the anti-interleukin-18 monoclonal antibody GSK1070806 [123] have shown safety and efficacy to inhibit IL-18 in human clinical trials and would be interesting to test in patients with high cardiovascular risk, alone or in combination with anti-IL-1β drugs, as IL-18 has been shown to correlate with major adverse cardiovascular events (MACEs) and for all-cause mortality in such patients [116]. Besides, repositioning of anti-IL-6 approved drugs, comprising anti-IL-6 receptor monoclonal antibodies (mAbs): tocilizumab and sarilumab and anti-IL-6 mAb and siltuximab [124], might be another useful strategy to lessen inflammation, and thus ameliorate vascular aging and its associated adverse outcomes.

Although most of the drugs directly targeting the NLRP3 inflammasome are still experimental, there are several approved drugs targeting one of its main products, IL-1β. Considering the recent trials showing decreased cardiovascular risk in patients with previous myocardial infarction and elevated high-sensitivity C-reactive protein (CRP) treated with canakinumab [7], a bright future can be envisioned for pharmacological agents directed to the NLRP3 inflammasome.
